# Kinesin family member 23, regulated by FOXM1, promotes triple negative breast cancer progression via activating Wnt/β-catenin pathway

**DOI:** 10.1186/s13046-022-02373-7

**Published:** 2022-05-07

**Authors:** Zhi Li, Hai-Yan Yang, Xiao-Lan Zhang, Xu Zhang, Yu-Zhou Huang, Xin-Yuan Dai, Liang Shi, Guo-Ren Zhou, Ji-Fu Wei, Qiang Ding

**Affiliations:** 1 Jiangsu Breast Disease Center, the First Affiliated Hospital with Nanjing Medical University, 300 Guangzhou Road, Nanjing, 210029 Jiangsu Province PR China; 2 Department of Breast and Thyroid Surgery, Huai’an First People’s Hospital, Nanjing Medical University, Huai’an, China; 3 Department of Breast Surgery, The Affiliated Changzhou No.2 People’s Hospital of Nanjing Medical University, #68 Gehu Middle Road, Wujin District, Changzhou, 213000 Jiangsu China; 4 Department of Oncology, Jiangsu Cancer Hospital & the Affiliated Cancer Hospital of Nanjing Medical University & Jiangsu Institute of Cancer Research, Nanjing, Jiangsu 210009 PR China; 5 Department of Pharmacy, Jiangsu Cancer Hospital & the Affiliated Cancer Hospital of Nanjing Medical University & Jiangsu Institute of Cancer Research, Nanjing, Jiangsu 210009 PR China; 6 Research Division of Clinical Pharmacology, the First Affiliated Hospital with Nanjing Medical University, 300 Guangzhou Road, Nanjing, 210029 Jiangsu Province PR China

**Keywords:** KIF23, Wnt/β-catenin pathway, FOXM1, WDR5, H3K4me3, Triple negative breast cancer

## Abstract

**Background:**

Triple negative breast cancer (TNBC) is highly malignant and has a worse prognosis, compared with other subtypes of breast cancer due to the absence of therapeutic targets. KIF23 plays a crucial role in the tumorigenesis and cancer progression. However, the role of KIF23 in development of TNBC and the underlying mechanism remain unknown. The study aimed to elucidate the biological function and regulatory mechanism of KIF23 in TNBC.

**Methods:**

Quantitative real-time PCR and Western blot were used to determine the KIF23 expression in breast cancer tissues and cell lines. Then, functional experiments *in vitro* and in *vivo* were performed to investigate the effects of KIF23 on tumor growth and metastasis in TNBC. Chromatin immunoprecipitation assay was conducted to illustrate the potential regulatory mechanisms of KIF23 in TNBC.

**Results:**

We found that KIF23 was significantly up-regulated and associated with poor prognosis in TNBC. KIF23 could promote TNBC proliferation, migration and invasion *in vitro* and *in vivo*. KIF23 could activate Wnt/β-catenin pathway and promote EMT progression in TNBC. In addition, FOXM1, upregulated by WDR5 via H3K4me3 modification, directly bound to the promoter of KIF23 gene to promote its transcription and accelerated TNBC progression via Wnt/β-catenin pathway. Both of small inhibitor of FOXM1 and WDR5 could inhibit TNBC progression.

**Conclusions:**

Our findings elucidate WDR5/FOXM1/KIF23/Wnt/β-catenin axis is associated with TNBC progression and may provide a novel and promising therapeutic target for TNBC treatment.

**Supplementary Information:**

The online version contains supplementary material available at 10.1186/s13046-022-02373-7.

## Background

Breast cancer is the most common malignant tumor in women and the leading cause of death among women [1, 2]. It is mainly divided into four subtypes: Luminal A, Luminal B, epidermal growth factor receptor (HER)2^+^ and triple negative breast cancer (TNBC) by the biomarkers estrogen receptor (ER), progesterone receptor (PR) and HER2. The TNBC makes up 10-20% of all breast cancers, lacking all of the biomarkers [3]. The prognosis of TNBC is significantly worse than other subtypes of breast cancer due to the absence of therapeutic targets [4]. It is urgent to discover new molecular targets to improve TNBC patients’ prognosis.

Kinesin superfamily (KIFs) consists of 45 family protein and is classified into 14 subfamilies. KIF23, which belongs to the kinesin superfamily [5], is a component of the central spindlin complex and involved in the cytokinesis [6–8]. It plays crucial roles in multiple normal cellular biological activities, such as cytoplasm separation in mitosis and cytokinesis [8, 9]. Previous studies demonstrated KIFs were involved in different types of cancers [5]. For example, KIF15 can promote cancer stem cell phenotype in hepatocellular carcinoma [10], while KIF18B can promote cell proliferation in hepatocellular carcinoma [11]. In addition, several studies have illustrated KIF23 participates in the occurrence of multiple cancers, including gastric cancer, bladder cancer, lung cancer and pancreatic ductal adenocarcinoma [12–16]. In gastric cancer, KIF23 promotes cancer cells proliferation via direct interaction with Amer1 [12]. In pancreatic ductal adenocarcinoma, overexpression of KIF23 predicts poor prognosis [15]. However, the functional role of KIF23 and the underlying mechanism in TNBC remain unknown.

Epithelial–mesenchymal transition (EMT) is associated with the tumorigenic process of many cancers [17]. Several studies have illustrated EMT is involved in TNBC progression [18, 19]. Moreover, accumulating evidence have demonstrated signaling pathways play crucial roles in many cancers, such as PI3K/AKT [20], JAK/STAT [21], Wnt/β-catenin [22], Hedgehog [23] signaling pathways and so on. Previous studies have illustrated that dysregulation of Wnt/β-catenin pathway is involved in different types of cancers [24–27]. Additionally, it is associated with TNBC proliferation and metastasis [28, 29] and may be a potential therapeutic target for TNBC treatment [30]. However, the molecular basis for the dysregulation of this pathway remains unclear.

In our study, we showed KIF23 was obviously upregulated in TNBC and downregulated KIF23 suppressed proliferation and metastasis of TNBC cells. In addition, downregulated KIF23 inhibited EMT progression and Wnt/β-catenin pathway in TNBC. Moreover, our results demonstrated FOXM1, upregulated by WDR5 via H3K4me3 modification, upregulated KIF23 expression via promoting KIF23 transcription in TNBC. Our findings elucidate WDR5/FOXM1/KIF23/Wnt/β-catenin axis is associated with TNBC progression and may provide a novel and promising therapeutic target.

## Material and methods

### Clinical tissue samples

Human TNBC tissues and paired adjacent normal tissues were collected from the First Affiliated Hospital of Nanjing Medical University. All patients received no neoadjuvant therapy. All tissues were frozen in liquid nitrogen immediately after resection and stored at -80 °C. The patients all provided written informed consent and the study was approved by the ethical committee of the First Affiliated Hospital of Nanjing Medical University.

### Bioinformatic analysis

The different expression genes in TNBC were analyzed by GEO2R with the datasets deposited in GEO. The expression of KIF23 in different subtypes of breast cancer was analyzed by UALCAN [31]. Relapse-free survival of TNBC was analyzed by Kaplan–Meier Plotter (https://kmplot.com/analysis/). CancerSEA and CGPE were used to analyze the biological function of KIF23 [32, 33]. Correlation between genes was analyzed by TIMER [34]. Cistrome was used to predict the transcription factor and histone modification [35]. CHIP-Seq data analysis and visualization were conducted by TFmapper [36].

### Cell lines and cell culture

All breast cancer cell lines including MCF-7, ZR-75-1, BT474, MDA-MB-231, MDA-MB-468, HCC1806 and normal human breast epithelial cell (MCF10A) were obtained from the American Tissue Culture Collection (ATCC, USA). SUM1315 cell line was provided by Stephen Ethier (University of Michigan). HCC1806 was cultured in RPMI-1640 medium (Gibco, USA), other cell lines were cultured in complete high glucose Dulbecco’s modified Eagle medium (DMEM) (Gibco, USA), containing 10% fetal bovine serum, 100 μg/ml penicillin-streptomycin (Hyclone, USA) at 37 °C with 5% CO_2_.

### RNA isolation and quantitative real time-polymerase chain reaction (qRT-PCR)

Total RNA was isolated using Trizol reagent (TaKaRa, Japan), and 1000 ng RNA was reverse-transcribed to cDNA using HiScript Q RT SuperMix (Vazyme, China). The qRT–PCR was performed using AceQ qPCR SYBR Green Master Mix (Vazyme, China) and GAPDH was used as endogenous control. The specific primers used are listed in Table S1.

### Western blot

Proteins were extracted and electrophoresed on a 10% sodium dodecyl sulfate polyacrylamide (SDS-PAGE) gels and transferred onto a polyvinylidene fluoride (PVDF) membrane (Millipore, USA). Then membranes were blocked in QuickBlock™ blocking buffer (Beyotime, China) for 20 min and incubated with primary antibodies overnight at 4 °C. The membranes were then incubated for 2 h in appropriate secondary antibodies after membranes were washed 10 min three times with Tris buffered saline Tween (TBST). The ECL chemiluminescent reagent (Millipore, USA) was used to visualize proteins

### Lentivirus transfection and small interfering RNAs

Cells were transfected with lentivirus (GenePharma, China) to downregulate KIF23 expression. Puromycin (3μg/ml) was used to select the stable cells for two weeks. The specific small interfering RNAs (siRNAs) for FOXM1 and WDR5 were purchased from GenePharma (China). The human KIF23 and FOXM1 overexpression pCMV3-KIF23 and pCMV3-FOXM1 vectors were obtained from Obio Technology (China). The sequences of siRNAs and shRNA were listed in Table S2.

### Cell proliferation assay

The Cell Counting Kit-8 (CCK-8; Vazyme, China) was used to detect cell proliferation according to the manufacturer’s protocol, and cell colony formation ability was assessed as described previously [37].

### 5-ethynyl-2′-deoxyuridine (EdU) assay

The cells were cultured in 96-well plates (2 × 10^4^ cells/well) for 24h, incubated with EdU at 37°C for 2h. And then, nuclei were stained with 4′,6-diamid-ino-2- phenylindole (DAPI), and the cells were visualized under a fluorescence microscope (Nikon, Japan).

### Immunofluorescence analysis (IF)

Stably transfected cells (sh-NC, sh-KIF23) were washed with PBS, and then fixed with Immunol Staining Fix Solution (Beyotime, China) for 10 min, washed with Immunol Staining Wash Buffer (Beyotime, China) three times, blocked with Immunol Staining Blocking Buffer (Beyotime, China) for 1h and incubated with α-tubulin primary antibodies (Abcam, USA) overnight at 4°C. The cells were washed three times and then incubated with anti-mouse antibody for 1 h. The cell nuclei were stained with DAPI for 10 min. Cells were captured with a fluorescence microscope (Nikon, Japan).

### Wound healing assay and transwell assay

Cell migration and invasion abilities were measured by the wound healing assay and transwell assay as reported previously [38].

### Chromatin immunoprecipitation assay (ChIP)

ChIP assays were performed using chromatin immunoprecipitation kits (17–371, EZ-ChIP, Millipore) according to the manufacturer’s instructions as in previous reports [39].

### Chemical reagents

FOXM1 inhibitor (Thiostrepton) and WDR5 inhibitor (OICR-9429) were purchased from MedChemexpress (Monmouth Junction, NJ, USA). The regents were dissolved in dimethyl sulfoxide (DMSO) to generate the stock solution. For functional assays, the stock solution was diluted with culture medium. The working concentrations of thiostrepton and OICR-9429 were 5μM and 10μM respectively.

### Animal study

All animal experiments were conducted according to the guidelines of Institutional Animal Care and Use Committee of the Nanjing Medical University. Twenty-four female BALB/c nude mice (aged 4 weeks, 18–22g) were randomly divided into 4 groups (MDA-MB-231-sh-NC, MDA-MB-231-sh-KIF23, SUM1315-sh-NC, SUM1315-sh-KIF23). Stable cells or control cells (1×10^6^ cells in 0.1 mL PBS) was subcutaneously injected into mammary fat pads of the mice and the growth of tumors was followed up every four days. For animal procedures of OICR-9429, 1×10^6^ wild type SUM1315 cells were subcutaneously injected into mammary fat pads of the mice and the mice bearing xenografts were randomly divided into 2 groups: (1) Control group, treated with PBS per 2 days; (2) OICR-9429 group, treated with OICR-9429 40mg/kg per 2 days. Tumor volume was measured every four days using a caliper, calculated as (length × width2)/2. After 20 days, mice were sacrificed and checked for final tumor weight. For metastasis assay, stable cells were injected into the tail vein of each mouse. After 8 weeks, mice were sacrificed and examined for lung metastasis.

### Immunohistochemistry (IHC) analysis

All samples were fixed and then embedded in paraffin. Then the sections were dried, de-waxed, rehydrated and incubated with the E-cadherin, N-cadherin, Vimentin and Ki-67 primary antibodies (Proteintech, USA) followed by secondary antibody conjugated with HRP. Finally, detection was conducted by 3,3′-diaminobenzidine and hematoxylin. The staining positivity was quantified in three different high-power fields of each section.

### Statistical analysis

Each experiment was repeated at least three times. The statistical analysis was performed using SPSS 24.0 and GraphPad Prism 8.0. Experimental data were shown as mean ± standard deviation (SD). The differences between groups were analyzed using Student’s t-test or ANOVA. *P* < 0.05 was considered statistically significant.

## Results

### KIF23 is upregulated in TNBC tissues and cell lines

**Fig. 1:**
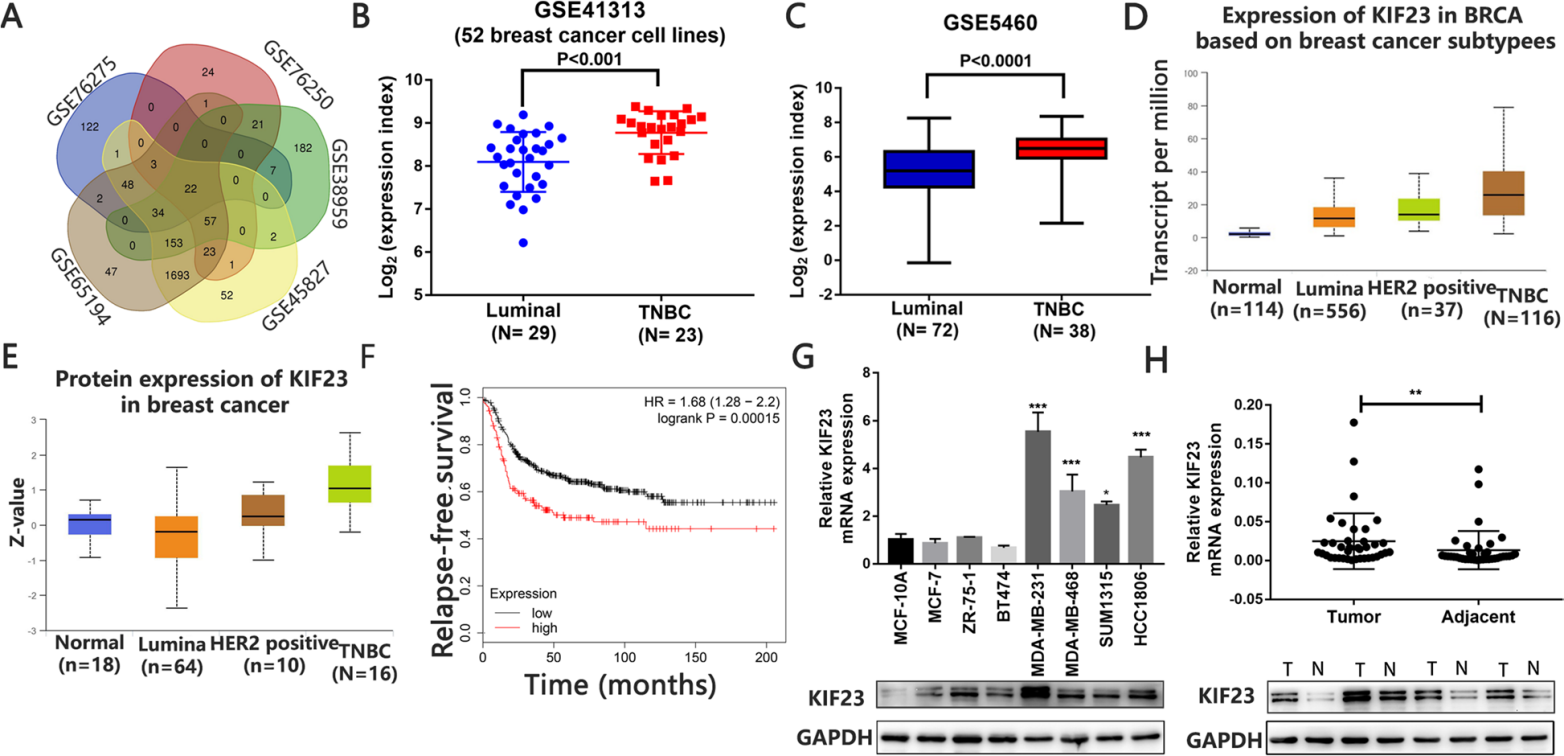
KIF23 is overexpressed in TNBC and associated with poor prognosis. **A** The Venn-diagram showed the upregulated genes in TNBC. **B** Expression of KIF23 in 52 breast cancer cell lines from public microarray dataset (GSE41313). **C** Expression of KIF23 in luminal breast cancer and TNBC from public microarray dataset (GSE5460). D-E: KIF23 mRNA **D** and protein **E** expression in different subtypes of breast cancer based on TCGA and CPTAC database. **F** Kaplan-Meier survival analysis showed the correlation between the expression of KIF23 and the relapse free survival of TNBC patients based on TCGA. **G** Expression of KIF23 in the breast cancer cell lines and MCF-10A. **H** Expression of KIF23 in TNBC tissues and adjacent normal tissues. The relative quantification was calculated by the 2^-ΔΔCt^ method and normalized based on GAPDH. Data were shown as mean ± SD. *p <0.05, **p <0.01, ***p<0.001

We first explored the different expression genes in TNBC using the published data deposited in GEO. KIF23 was significantly up-regulated in TNBC (Fig. 1A and Fig. S1A-E). We then analyzed a public microarray dataset (GSE41313) including gene expression data of 52 kinds of breast cancer cell lines. The result showed KIF23 was significantly upregulated in TNBC cell lines compared with luminal cell lines (Fig. 1B). The findings were consolidated by the analysis of two other microarray datasets (GSE5460, GSE1456) and TCGA data (Fig. 1C-E and Fig. S1F). Moreover, patients with low KIF23 expression showed better prognosis (Fig. 1F). KIF23 expression was also determined in different subtypes of breast cancer cell lines and normal human breast cell lines by qRT-PCR and western blot analysis. However, KIF23 mRNA expression was only highly expressed in TNBC cell lines, compared to the human normal breast cell line. While protein levels were highly expressed in all tumor cell lines (Fig. 1G). We next detected KIF23 expression in 38 paired tumor tissues and in corresponding adjacent tissues from TNBC patients. The results showed KIF23 was significantly up-regulated in TNBC tissues (Fig. 1H).

### KIF23 promotes TNBC cells proliferation

**Fig. 2:**
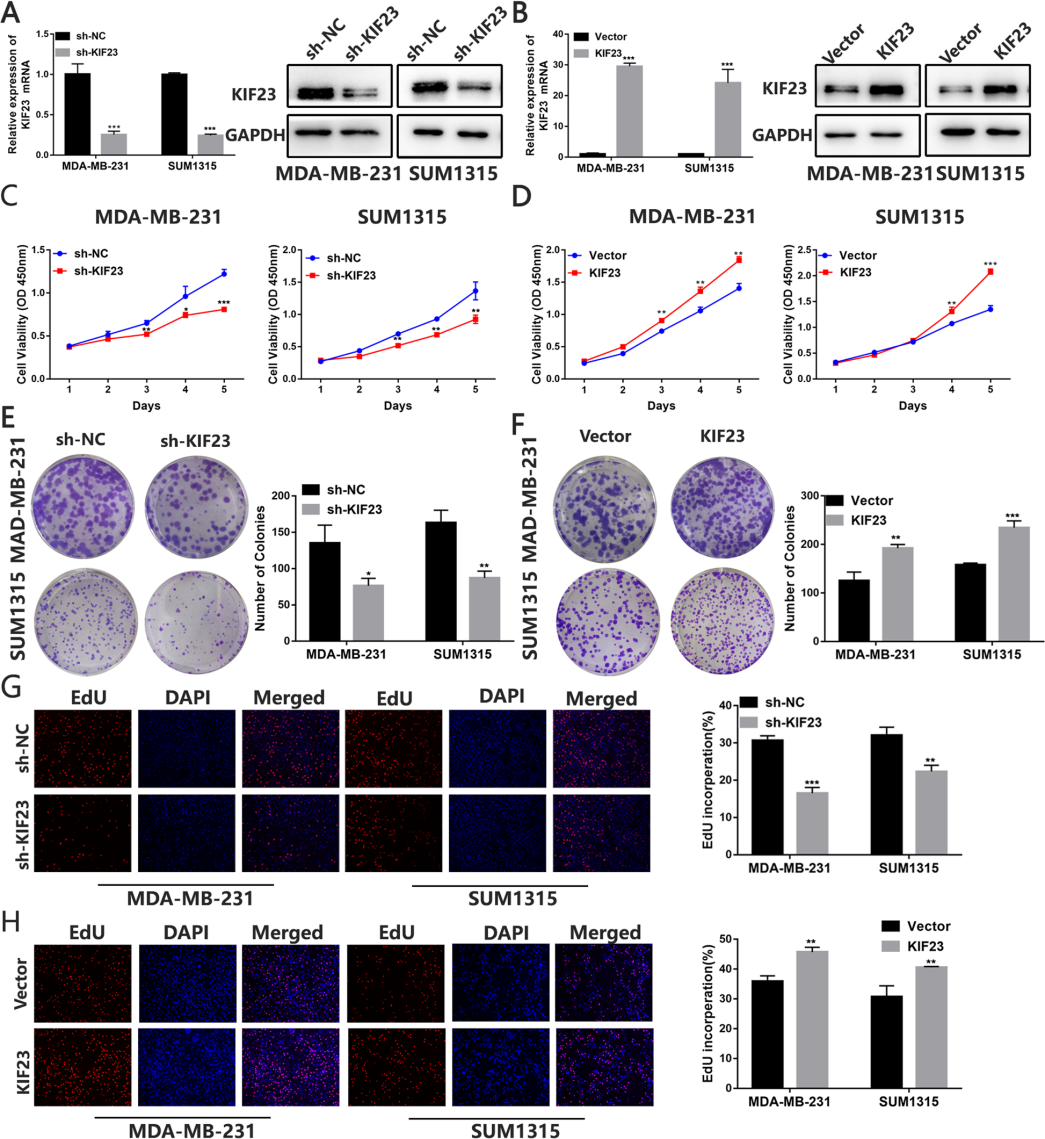
KIF23 promotes the proliferation of TNBC cells. **A-B** qRT-PCR and western blot were used to verify the efficiency of KIF23 knockdown **A** and overexpression **B** in MDA-MB-231 and SUM1315 cells. **C-D** Cell proliferation was determined by CCK-8 assays in MDA-MB-231 and SUM1315 cells after knockdown **C** or overexpression **D** of KIF23. **E-F** The colony formation results of MDA-MB-231 and SUM1315 cells after knockdown **E** or overexpression **F** of KIF23. Colonies > 50 mm were counted. **G-H** EdU assays results of MDA-MB-231 and SUM1315 cells after knockdown **G** or overexpression **H** of KIF23. Blue indicates DAPI, red indicates EdU. Data were shown as mean ± SD. *p <0.05, **p <0.01, ***p<0.001

We performed KEGG pathway analysis on co-expressing genes with KIF23, and found that these co-expressing genes were mainly enriched in the cell cycle pathway (Fig. S2A and Table S1). In addition, by analyzing a single cell database, we found that KIF23 was associated with cell cycle and proliferation in a variety of tumors (Fig. S2B). In breast cancer datasets, KIF23 expression was significantly related to cell cycle and proliferation, which was confirmed by several datasets (Fig. S2C). Moreover, the expression of KIF23 in patients without lymph node metastasis was significantly lower than that in patients with lymph node metastasis (Fig. S2D). However, the association was only detected in Luminal subtype (Fig. S2E-F). We also performed GSEA analysis after grouping TCGA patients according to the expression of KIF23 and found KIF23 might activate cell cycle pathway (Fig. S2G-H). Cells were transfected with lentiviral vectors containing shRNA-targeting KIF23 (sh-KIF23) or KIF23 overexpression plasmids (KIF23) respectively, with the efficiency confirmed by qRT-PCR and western blot analysis (Fig. 2A-B). The growth curves derived from the CCK-8 assay showed that the cell proliferation rate was significantly reduced after the cells were transfected with sh-KIF23 while cell proliferation was increased in response to KIF23 upregulation (Fig. 2C-D). Similarly, colony formation assay indicated clonality of the TNBC cells was suppressed by KIF23 downregulation but markedly increased by KIF23 overexpression (Fig. 2E-F). The EDU assay results indicated downregulated KIF23 inhibited cell DNA replication ability while overexpression of KIF23 caused reverse effects (Fig. 2G-H). Moreover, we demonstrated knockdown of KIF23 could cause mitotic defects in TNBC cells, which was reported to be associated with kinesins’ functions (Fig. S3).

### KIF23 facilitates TNBC cell invasion and migration

**Fig. 3:**
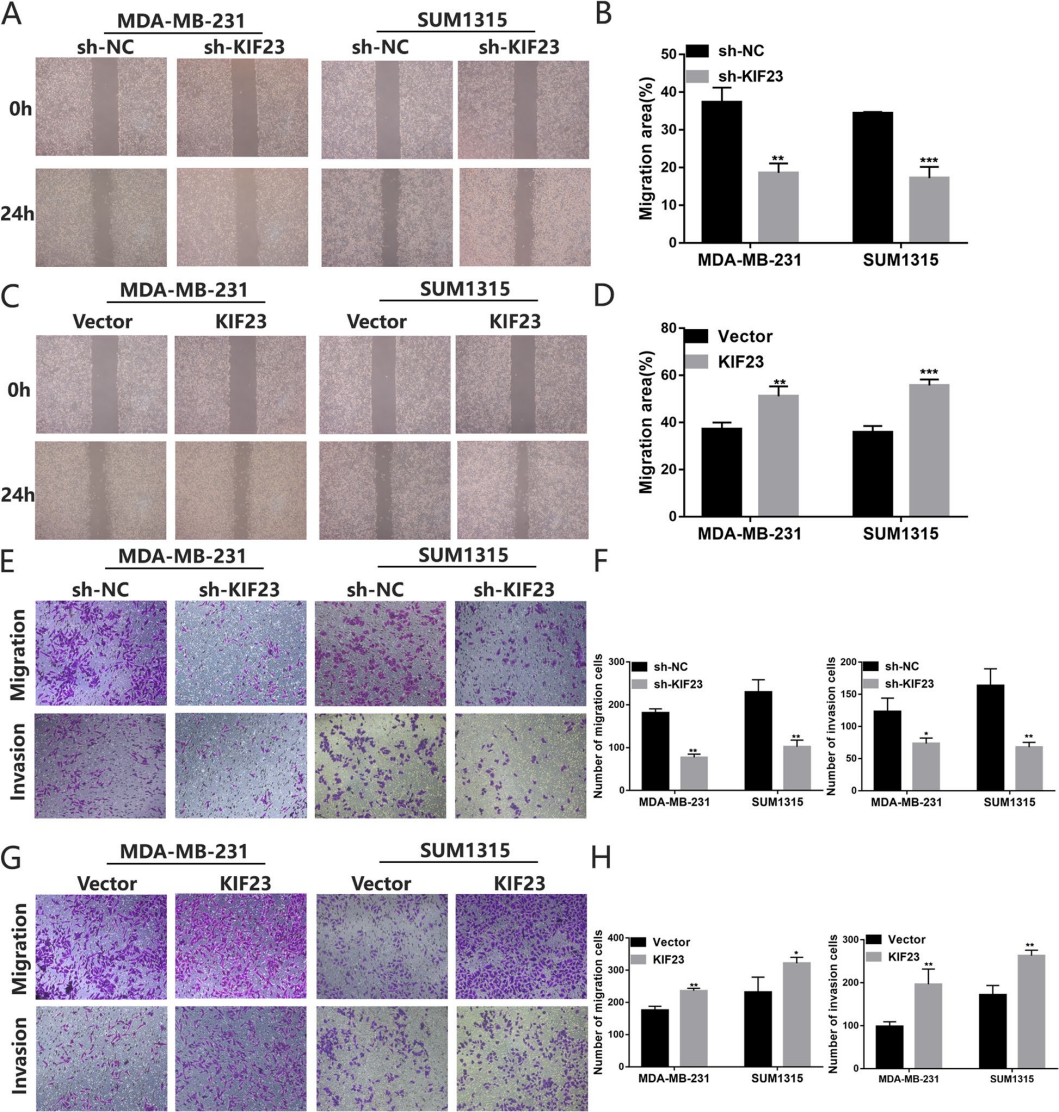
KIF23 enhances the migration and invasion of TNBC cells. **A-D** The wound healing assays were performed to assess the effect of knockdown **A-B** or overexpression **C-D** of KIF23 on cell motility at 0 and 24h in MDA-MB-231 and SUM1315 cells. **E-H** The representative images of migrated and invaded MDA-MB-231 and SUM1315 cells after KIF23 knockdown **E-F** or overexpression **G-H**. Data were shown as mean ± SD, *p <0.05, **p <0.01, ***p<0.001

In the wound healing assay, the migration rate was dramatically inhibited in KIF23 knockdown group but overexpression KIF23 enforced TNBC cell migration rate (Fig. 3A-D). In the transwell assay, the number of migration and invasion cells was decreased after the downregulation of KIF23 expression (Fig. 3E-F), while overexpression KIF23 caused the reversed effect (Fig. 3G-H).

### KIF23 promotes the epithelial–mesenchymal transition and Wnt/β-catenin signaling pathway in TNBC

**Fig. 4:**
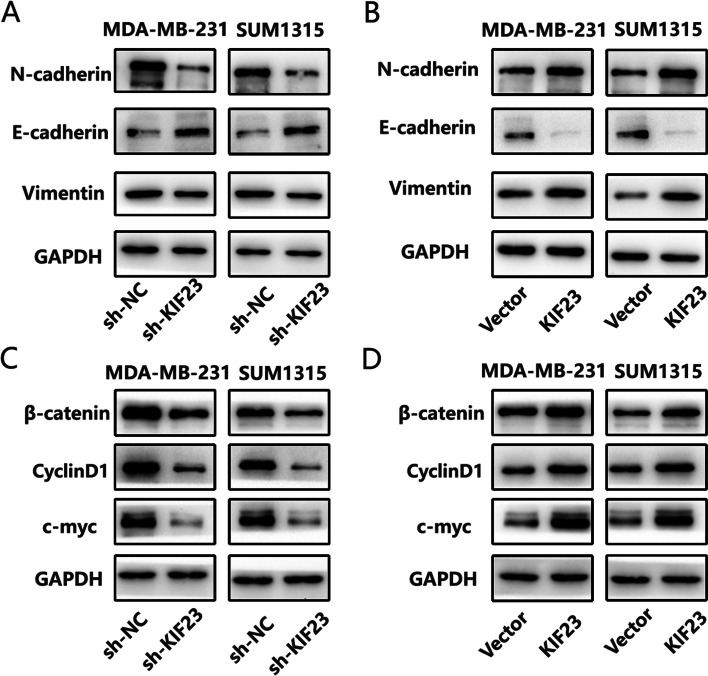
KIF23 enhances EMT progression and activates Wnt/β-catenin signaling pathway. **A-B** Western blot was used to detect protein level of biomarkers of EMT in MDA-MB-231 and SUM1315 cells after knockdown **A** or overexpression **B** of KIF23. **C-D** Western blot was used to detect protein level of biomarkers of Wnt/β-catenin signaling pathway in MDA-MB-231 and SUM1315 cells after knockdown **C** or overexpression **D** of KIF23

Previous studies showed EMT progression plays a crucial role in TNBC [18, 40, 41]. We detected EMT marker proteins by western blot. The results showed knockdown of KIF23 decreased N-cadherin and vimentin levels and increased E-cadherin level (Fig. 4A), while overexpression of KIF23 caused the opposite effects (Fig. 4B). Meanwhile, downregulated KIF23 decreased Wnt/β-catenin signaling pathway related genes expression, while upregulated KIF23 reversed this effect (Fig. 4C-D).

### KIF23 expression is directly regulated by FOXM1

**Fig. 5:**
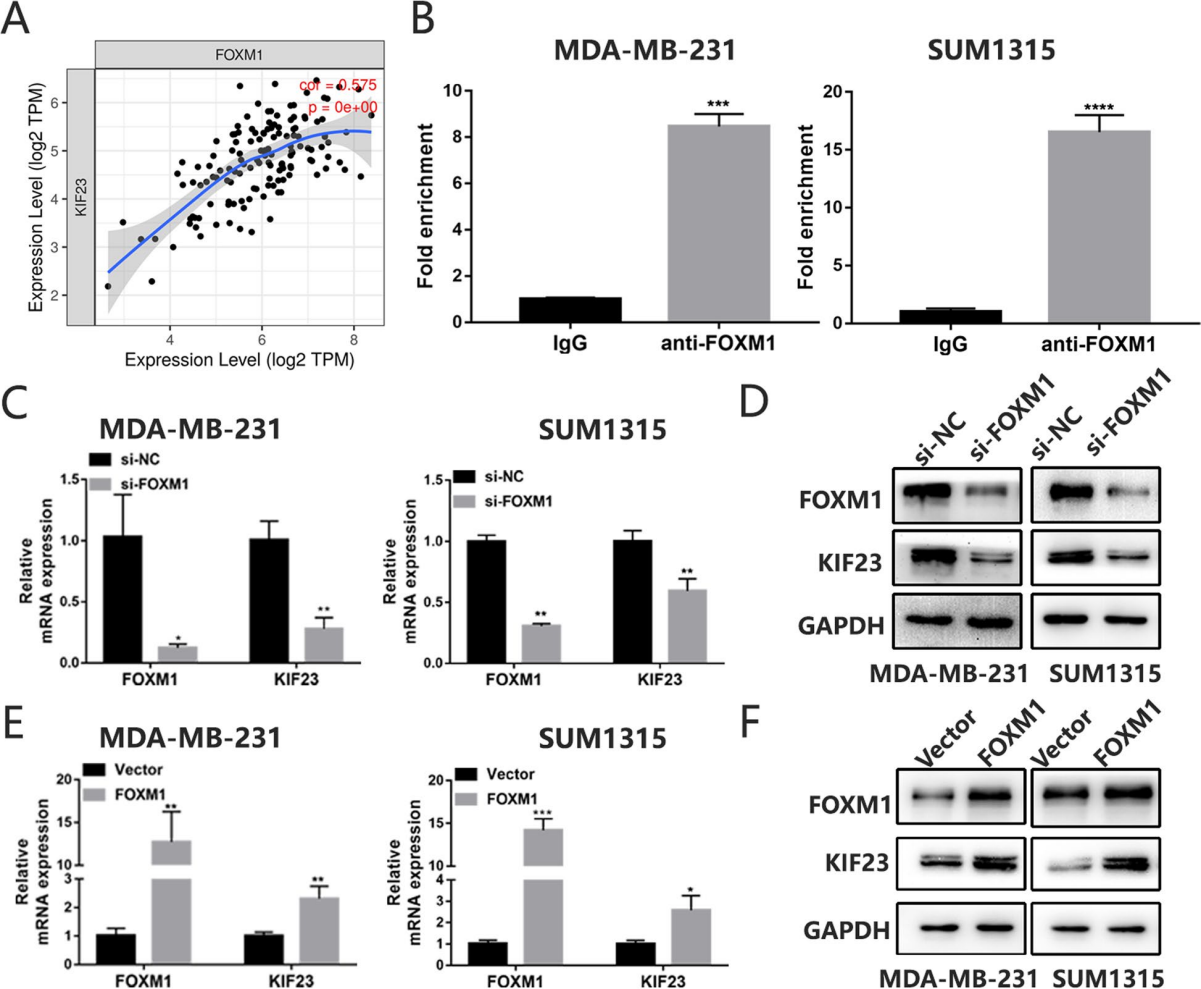
FOXM1 directly binds to KIF23 promoter to regulate KIF23 expression. **A** The correlation between expression of FOXM1 and KIF23. **B** CHIP assays were performed in MDA-MB-231 and SUM1315 cells. **C-F** qRT-PCR and western blot were used to verify the effect of FOXM1 knockdown **C-D** or overexpression **E-F** on KIF23 expression in MDA-MB-231 and SUM1315 cells. Data were shown as mean ± SD, *p <0.05, **p <0.01, ***p<0.001

As transcription factors (TFs) play crucial roles in cancers [42]. We predicted TFs that might regulate the KIF23 expression by bioinformatics methods. According to the prediction results, we analyzed the correlation between top 5 TFs and KIF23 expression in TNBC (Fig. 5A and Fig. S4A-B). The result showed the expression of Foxm1 and KIF23 were the most relevant. Then we analyzed the motif of FOXM1 binding and whether it existed in the promoter region of KIF23, the result demonstrated there was a binding peak of FOXM1 in the promoter region of KIF23 (Fig. S4C-D). The CHIP analysis also marked the FOXM1 occupancy at KIF3 promoter (Fig. 5B). In addition, the KIF23 expression was detected in FOXM1 knockdown or overexpression TNBC cell lines by qRT-PCR and Western blot. KIF23 expression was decreased after knockdown of FOXM1, while FOXM1 overexpression reversed the effect (Fig. 5C-F). In conclusion, these results illustrated FOXM1 could bind to KIF23 promoter region to promote KIF23 expression.

### FOXM1 enhance TNBC cells proliferation, migration and invasion by regulating KIF23 expression

**Fig. 6:**
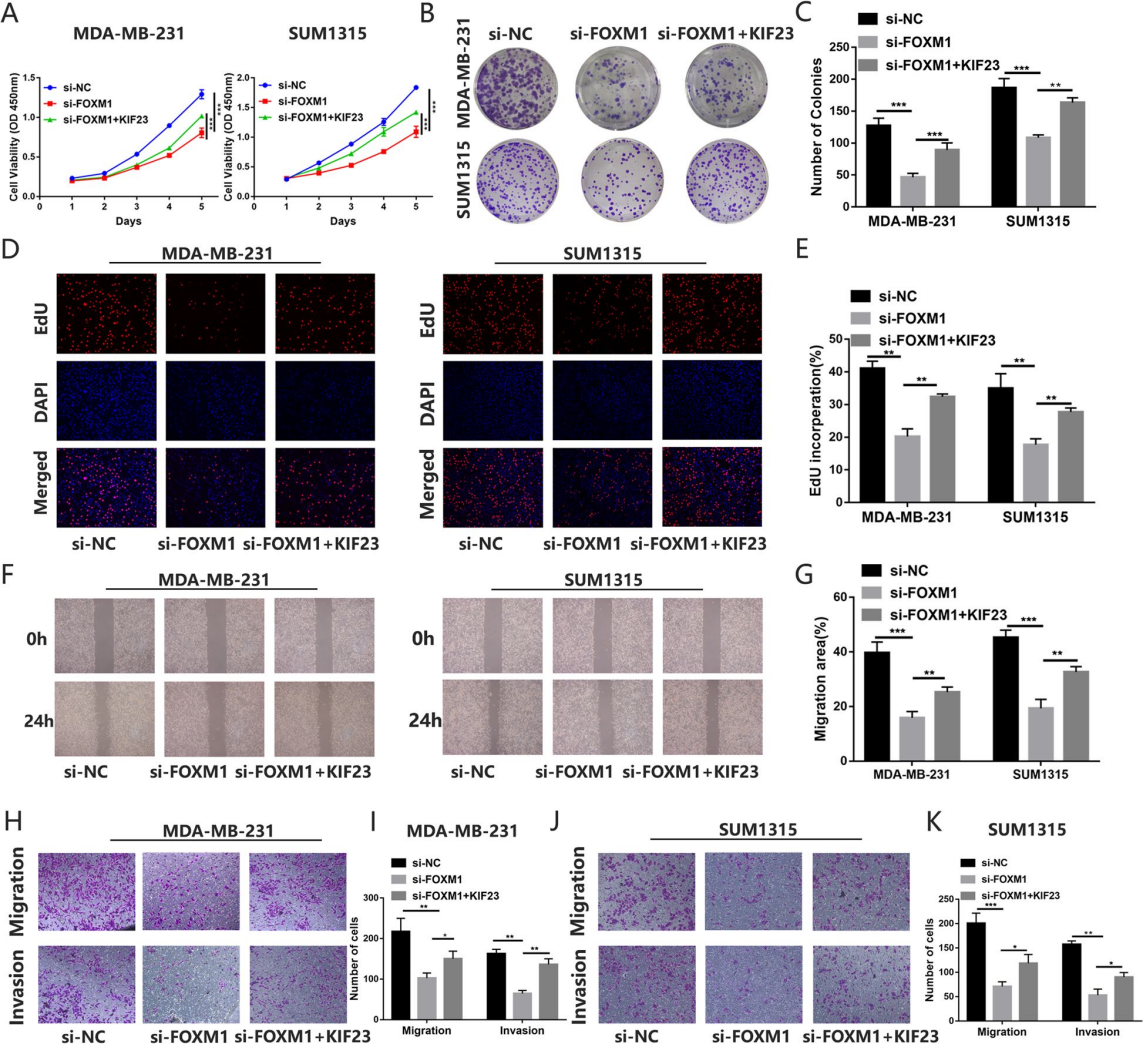
KIF23 partly restores the effect caused by FOXM1 in TNBC cells. **A-E** CCK-8 assays **A** colony formation assays **B-C** and EdU assays **D-E** indicated KIF23 could partly rescue the repressive effect of knockdown of FOXM1 on cell proliferation. **F-K** The wound healing assays **F-G** and transwell assays **H-K** demonstrated the overexpression of KIF23 partly reversed the repressive effect on cell migration and invasion caused by knockdown of FOXM1. Data were shown as mean ± SD, *p <0.05, **p <0.01, ***p<0.001

Previous studies showed FOXM1 plays a crucial role in TNBC and acts as a specific marker for TNBC. Downregulated FOXM1 by tansfecting siRNA or using thiostrepton inhibited TNBC cells proliferation, migration and invasion (Fig. S5). We next explored whether KIF23 was involved in the function of FOXM1 in TNBC cells. We found overexpression of KIF23 could partly reverse the decrease in breast cancer cells proliferation caused by the knockdown of FOXM1 (Fig. 6A-E). Moreover, overexpression of KIF23 could promote TNBC cells migration and invasion abilities inhibited by the downregulated FOXM1 (Fig. 6F-K). These results suggested KIF23 was the target of FOXM1in promoting TNBC progression.

### WDR5 upregulates FOXM1 expression by accelerating H3K4me3 modification

**Fig. 7:**
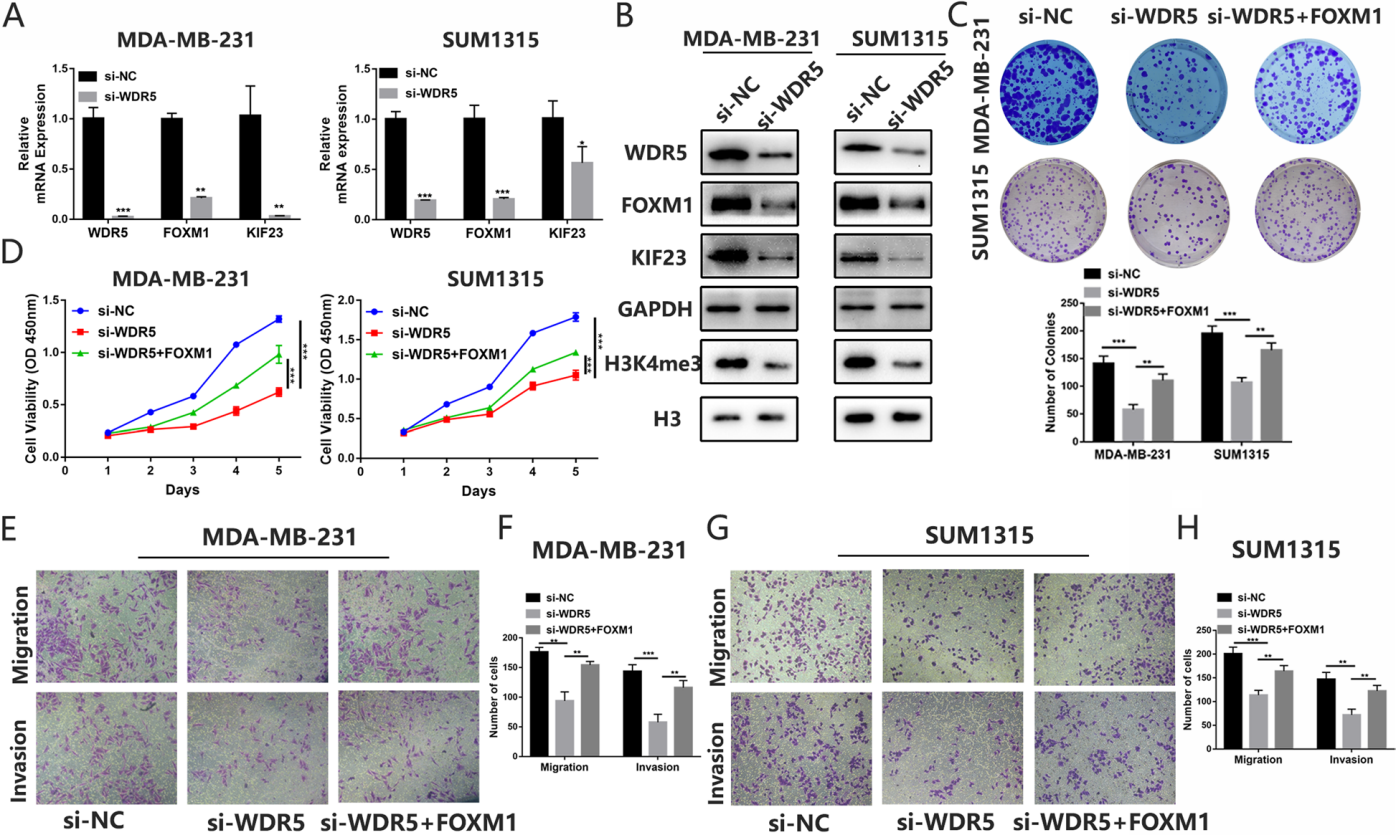
WDR5 regulates TNBC cells biological function via regulating FOXM1. **A** qRT-PCR was used to verify the expression of WDR5, FOXM1 and KIF23 after transfected with si-WDR5. **B** Western blot was used to verify the expression of WDR5, FOXM1, KIF23 and H3K4me3 after transfected with si-WDR5. **C-D** CCK-8 assays **C** and colony formation assays indicated FOXM1 could partly rescue the repressive effect of knockdown of WDR5 on cell proliferation. **E-H** The transwell assays demonstrated the overexpression of FOXM1 partly reversed the repressive effect on cell migration and invasion caused by knockdown of WDR5 in MDA-MB-231 **E-F** and SUM1315 **G-H**. Data were shown as mean ± SD, *p <0.05, **p <0.01, ***p<0.001

Histone modification plays an important role in the regulation of gene expression [43]. We explored whether histone modification affected FOXM1 expression. Through bioinformatics analysis, we found H3K4me3, which was reported to promote genes expression [44], might affect the FOXM1 expression (Fig. S6A). We then analyzed the correlation between components of methylation complex and FOXM1 expression in TNBC. The result showed the expression of Foxm1 and WDR5 were the most relevant (Fig. S5B-F). Public CHIP data showed a binding peak of H3K4me3 in the promoter region of FOXM1 and the CHIP analysis illustrated marked WDR5 and H3K4me3 occupancy at FOXM1 promoter (Fig. S6G-I). Moreover, knockdown of WDR5 inhibited FOXM1 and H3K4me3 expression (Fig. 7A-B). Downregulated WDR5 by transfecting siRNA or using OICR-9429, a small-molecule antagonist of the WDR5-MLL interaction, inhibited TNBC cells proliferation, migration and invasion (Fig. S7). We next explored whether FOXM1 was involved in the function of WDR5 in TNBC cells. The results demonstrated overexpression of FOXM1 partly reversed the decrease in breast cancer cells proliferation caused by knockdown of WDR5 (Fig. 7C-D). Meanwhile, overexpression rescued the inhibition of TNBC migration and invasion abilities caused by downregulated WDR5 (Fig. 7E-H). These results illustrated WDR5 promoted TNBC progression via upregulating FOXM1 expression.

### KIF23 promotes TNBC proliferation and metastasis *in vivo*

**Fig. 8:**
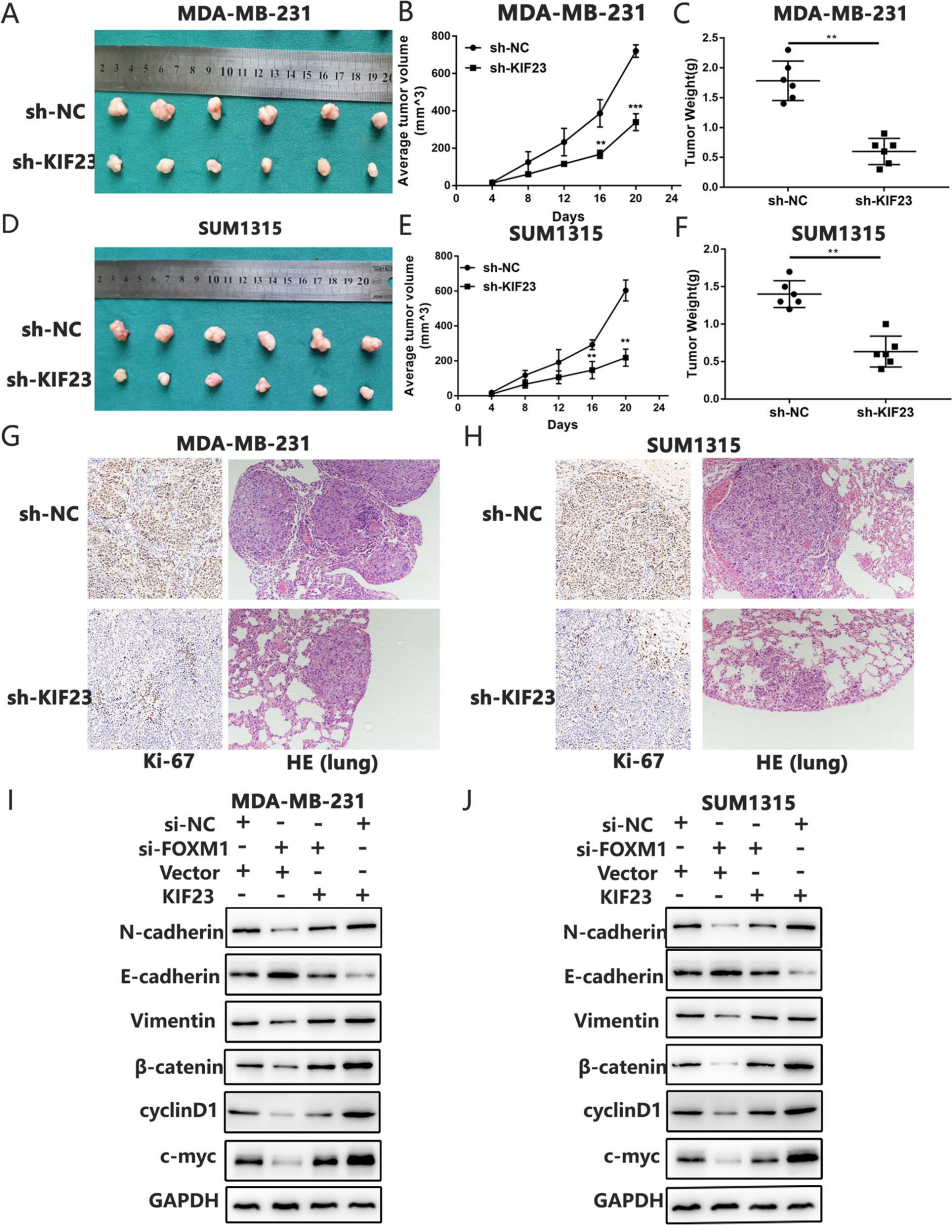
Knockdown of KIF23 inhibits TNBC progression in vivo by targeting Wnt/β-catenin signaling pathway. **A-F** Representative images of xenograft tumors, tumor sizes and weights of KIF23 knockdown (sh-KIF23) and control (sh-NC) group. **G-H** Ki-67 expression was analyzed by IHC and lung metastasis nodes were analyzed by H&E. **I-J** Western blot was used to determine the protein expression of genes that related to the pathways after transfecting with si-FOXM1 and protein expression level of these genes was detected after the restoration of KIF23. Data were shown as mean ± SD, *p <0.05, **p <0.01, ***p<0.001

To further investigate the role of KIF23 in *vivo*, stable cell lines were injected subcutaneously into BALB/c nude mice. As shown in Fig. 8A-F, tumor sizes and weights of KIF23 knockdown group were significantly lower than those of the control group. Moreover, we analyzed Ki-67 and EMT markers expression by IHC and lung metastasis by H&E. The results indicated knockdown of KIF23 alleviated tumor growth and metastasis in *vivo* (Fig. 8G-H and Fig. S8). Previous studies have demonstrated OICR-9429 could enhance chemosensitivity and inhibit tumor progression in bladder cancer and prostate cancer *in vivo* [45, 46], so we then explored the antitumor capacity of OICR-9429 in TNBC *in vivo.* As shown in Fig. S9, treatment of OICR-9429 could inhibit TNBC progression *in vivo*, indicating OICR-9429 might be used to treat TNBC.

### FOXM1 regulates the EMT progression and Wnt/β-catenin pathway via KIF23

**Fig. 9:**
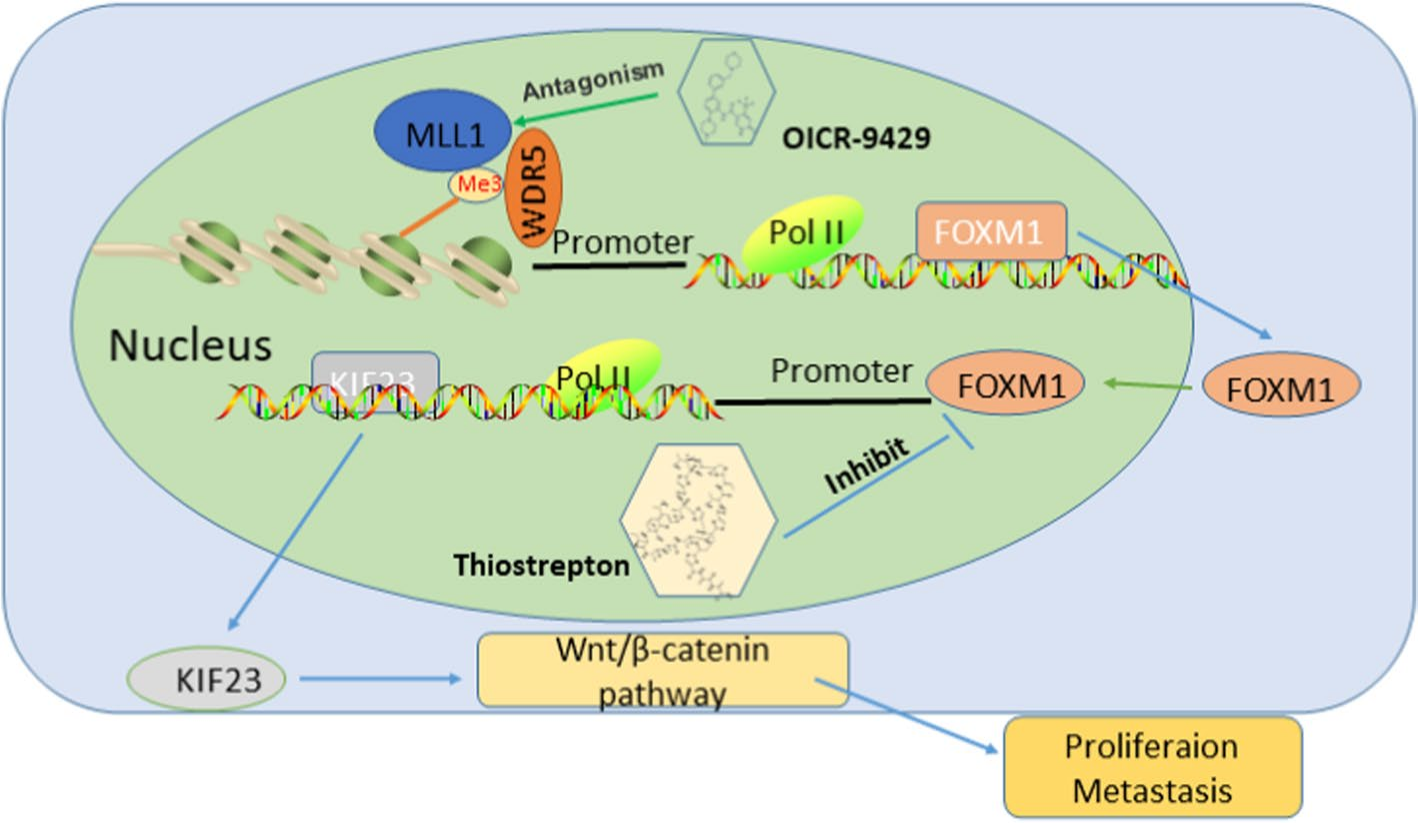
Molecular mechanism of WDR5/FOXM1/KIF23/Wnt/β-catenin axis in the progression in TNBC

As knockdown of KIF23 inhibited EMT progression and β-catenin pathway, we explored whether these effects were regulated by FOXM1. Moreover, downregulated FOXM1 decreased the expression of EMT and Wnt/β-catenin pathway related genes, but overexpression of KIF23 could reverse the effect (Fig. 8I-J). Altogether, FOXM1, upregulated by WDR5 via H3K4me3 modification, regulated the EMT progression and Wnt/ β-catenin pathway by regulating KIF23 expression (Fig. 9).

## Discussion

TNBC is a subtype of breast cancer with high heterogeneity, high recurrence, early metastasis and poor prognosis [4]. Due to the lack of hormone receptors and HER-2, endocrine therapy, targeted therapy and chemotherapy often fail to be effective treatments for TNBC [47, 48]. It is urgent to find potential molecular targets to improve the prognosis of TNBC. In our study, we found KIF23 was upregulated in TNBC and associated with prognosis of TNBC by bioinformatic analysis. Several studies showed KIF23 plays an oncogenic role in several cancer types [13, 49]. Moreover, our results illustrated KIF23 was upregulated in TNBC cell lines and tissues. Knockdown of KIF23 inhibited TNBC proliferation and metastasis while upregulated KIF23 caused opposite effects. Meanwhile, IF results showed knockdown of KIF23 could cause mitotic defects in TNBC cells, which was reported to be associated with kinesins’ functions. Previous studies have demonstrated Wnt/β-catenin signaling pathway is involved in different types of tumors progression [22, 50], including breast cancer [51, 52], gastric cancer [13], hepatocellular carcinoma [53], lung cancer [54]. And aberrant Wnt signaling pathway was associated with tumor proliferation, metastasis, stemness and drug resistance [25, 26, 55, 56]. It was reported KIF23 could activate Wnt/β-catenin signaling pathway in gastric and colorectal cancer [13, 49], and then we explored whether KIF23 affected Wnt/β-catenin signaling pathway in TNBC. Our results showed the related genes of Wnt/β-catenin signaling pathway, such as β-catenin, CyclinD1 and c-myc, were upregulated due to the KIF23 overexpression, indicating KIF23 promotes TNBC progression via activating this pathway. It was reported KIF23 could precipitate with protein regulator of cytokinesis 1 (PRC1) [13, 57], which could promote β-catenin stabilisation by suppressing adenomatous polyposis coli (APC) function [58]. We suspected KIF23 might affect Wnt/β-catenin pathway through regulating β-catenin stability. In addition, Wnt/β-catenin signaling pathway can promote EMT progression [53], which is associated with tumor progression [25, 54]. We also found knockdown of KIF23 decreased EMT marker proteins expression, suggesting KIF23 could promote EMT progression in TNBC.

FOXM1 is a transcription factor and plays a crucial role in different types of cancers, including gastric cancer, lung cancer and prostate cancer [59–62]. In breast cancer, FOXM1 was identified as a specific marker for TNBC and enhanced paclitaxel resistance in TNBC [63, 64]. In the present study, we found FOXM1 might be recruited and bind to the promoter region of KIF23 via analyzing public CHIP-Seq datasets and FOXM1 expression is positively correlated with KIF23 expression in TNBC tissues. Inhibition of FOXM1 with siRNA or thiostrepton [65] significantly suppressed TNBC cells proliferation and migration, while overexpression of KIF23 could partly rescue the effects caused by FOXM1 inhibition. Meanwhile, FOXM1 could promote EMT progression and activate Wnt/β-catenin pathway, which is in accord with previous study [66]. Thus, FOXM1 could accelerate TNBC progression via regulating transcriptional activity of KIF23.Additionaly, thiostrepton, the selective inhibitor of FOXM1, might be the potential therapy drug for TNBC.

Epigenetics refers to the reversible and heritable changes in gene expression and function without changes in the DNA sequence, including DNA modification, various modifications of histones, and so on [67]. H3K4me3 modification is a common modification of histones, which affects transcription of genes [43]. In the present study, we detected histone modification of FOXM1 by bioinformatics analysis and found H3K4me3 modification might affect FOXM1 expression. Subsequent experiments demonstrated WDR5 upregulated FOXM1 expression via promoting FOXM1 H3K4me3 modification. In addition, the effects caused by inhibition of WDR5 on proliferation and metastasis of TNBC cells could be partly restored by the upregulating FOXM1 expression. Our results illustrated WDR5 increased H3K4me3 modification to upregulate FOXM1 expression. Knockdown of WDR5 or small molecule antagonist of WDR5-MLL complex could significantly inhibit TNBC cells proliferation and metastasis, suggesting OICR-9429 might be used to treat TNBC.

## Conclusion

In conclusion, our results demonstrated KIF23 plays a crucial role in TNBC progression. Mechanistically, KIF23 promotes TNBC proliferation and metastasis abilities via activating Wnt/β-catenin pathway and promoting EMT progression. Meanwhile, FOXM1, upregulated by WDR5 via H3K4me3 modification, promotes KIF23 expression through enhancing transcription of KIF23. Our findings elucidate WDR5/FOXM1/KIF23/Wnt/β-catenin axis is associated with TNBC progression and may provide a novel and promising therapeutic target. Accordingly, thiostrepton and OICR-9429 alone or in combination have the potential to treat TNBC.

## Supplementary Information

**Additional file 1: Figure S1.** Expression of KIF23 in GEO datasets.

**Additional file 2: Figure S2.** KIF23 participates in breast cancer proliferation and metastasis.

**Additional file 3: Figure S3.** Knockdown of KIF23 causes mitotic defects in TNBC cells.

**Additional file 4: Figure S4.** FOXM1 regulates KIF23 by binding the promoter region of KIF23.

**Additional file 5: Figure S5.** Inhibition of FOXM1 represses TNBC cell proliferation, migration and invasion.

**Additional file 6: Figure S6.** WDR5 regulates FOXM1 expression via H3K4me3 modification.

**Additional file 7: Figure S7.** Inhibition of WDR5 represses TNBC cell proliferation, migration and invasion.

**Additional file 8: Figure S8.** Knockdown of KIF23 reduces EMT markers and Ki-67 expression *in vivo.*

**Additional file 9: Figure S9.** OICR-9429 treatment inhibits TNBC progression *in vivo.*

**Additional file 10: Table S1.** The primer sequence of qRT-PCR and CHIP analysis. **Table S2.** siRNA and RNA oligonucleotides sequences.

## Data Availability

The datasets generated during and/or analysed during the current study are available in Gene Expression Omnibus (GEO) database.

## Abbreviations

TNBC: Triple negative breast cancer
KIF23: Kinesin family member 23
TCGA: The Cancer Genome Atlas
FOXM1: Forkhead Box M1
WDR5: WD Repeat Domain 5
EMT: Epithelial–mesenchymal transition
qRT-PCR: RNA isolation and quantitative real time-polymerase chain reaction
CCK8: Cell Counting Kit-8
EdU: 2.8 5-ethynyl-2′-deoxyuridine
CHIP: Chromatin immunoprecipitation assay
