# Supplementary figures and images for: Kinesin family member 23, regulated by FOXM1, promotes triple negative breast cancer progression via activating Wnt/β-catenin pathway
Source: J Exp Clin Cancer Res. 2022 May 7;41:168. doi: 10.1186/s13046-022-02373-7 (PMC9077852; doi:10.1186/s13046-022-02373-7)

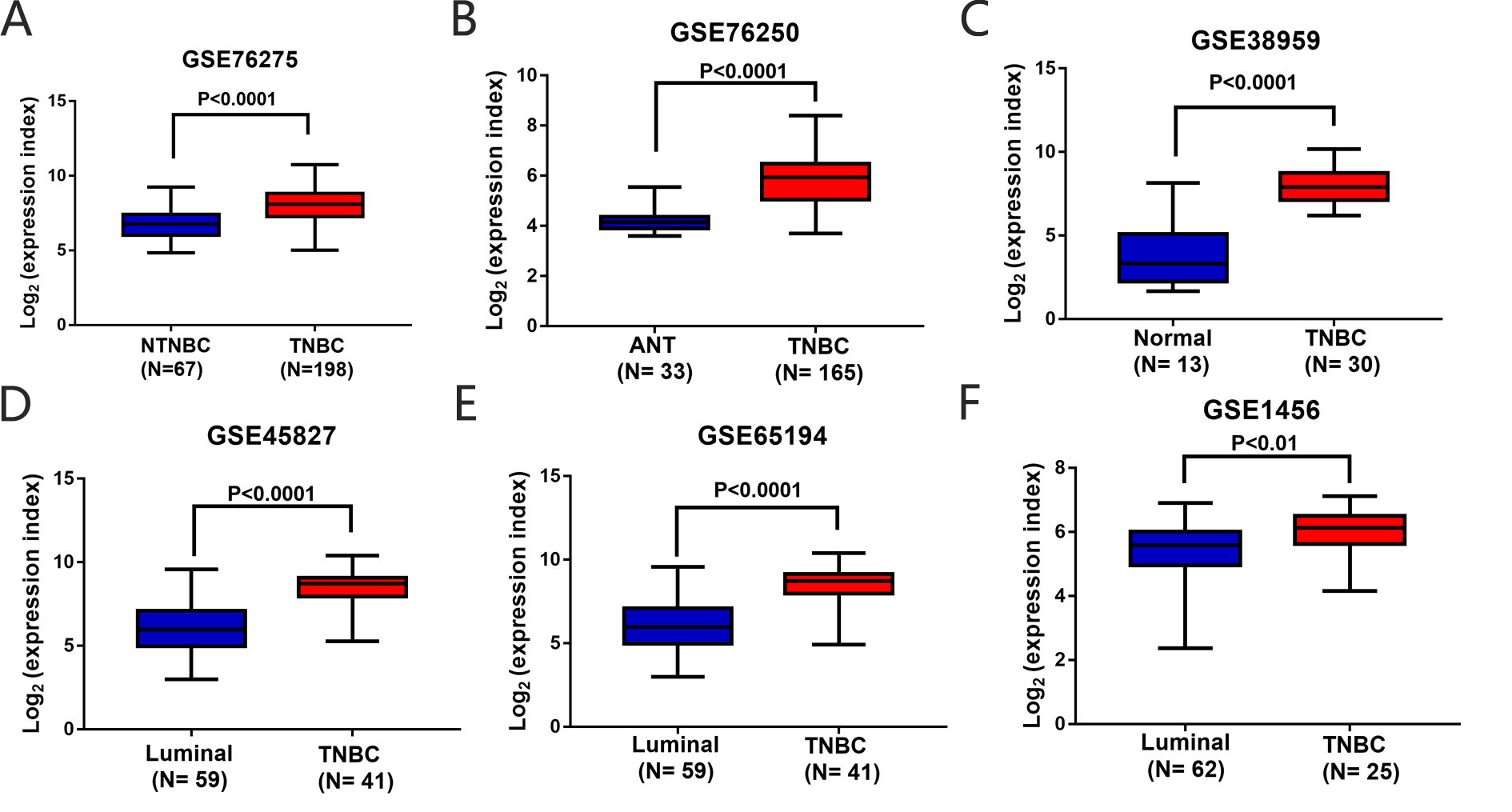

Supplement: Supplementary file 1 — Additional file 1: Figure S1. Expression of KIF23 in GEO datasets. [file 13046_2022_2373_MOESM1_ESM.tif]

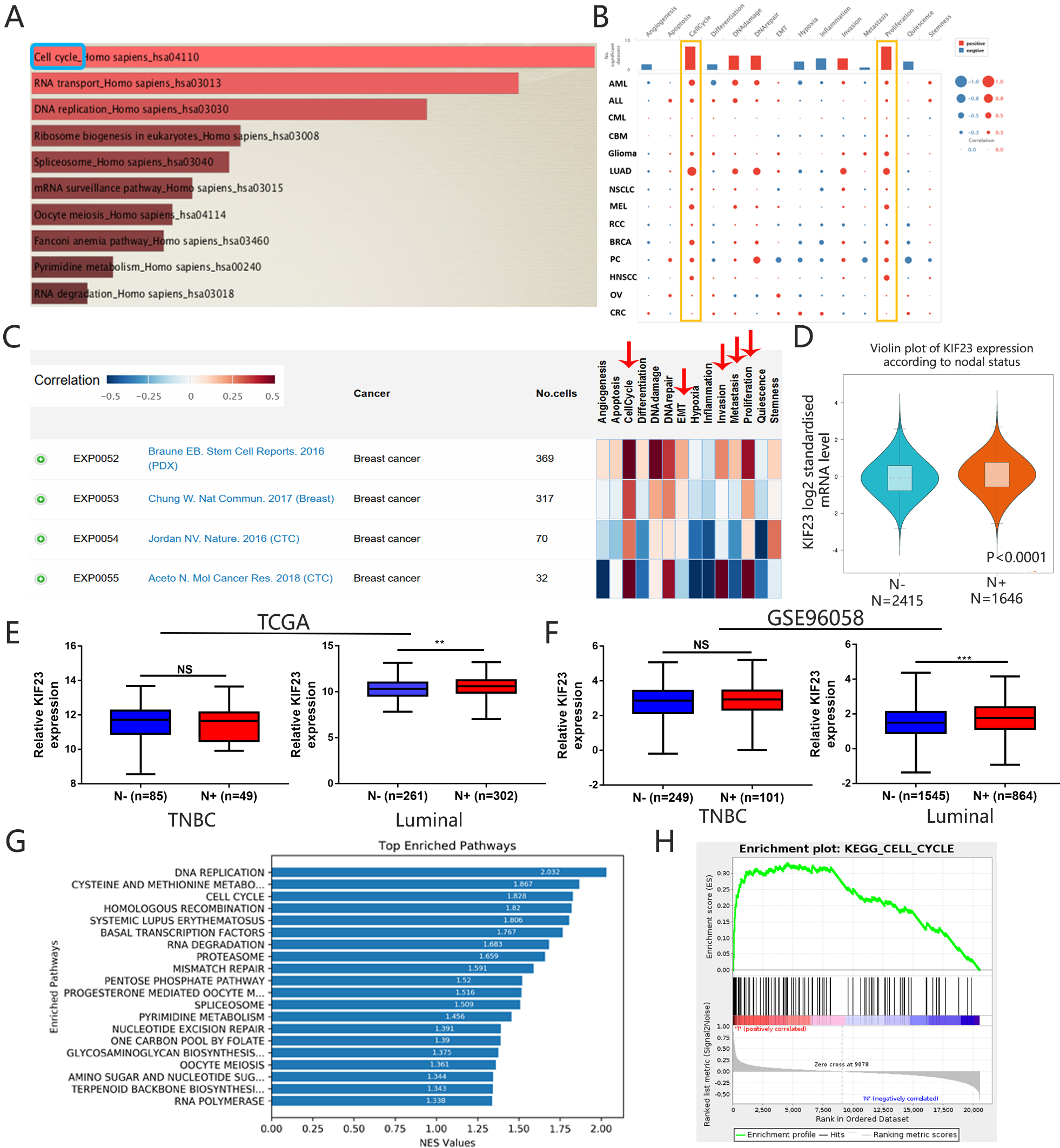

Supplement: Supplementary file 2 — Additional file 2: Figure S2. KIF23 participates in breast cancer proliferation and metastasis. [file 13046_2022_2373_MOESM2_ESM.tif]

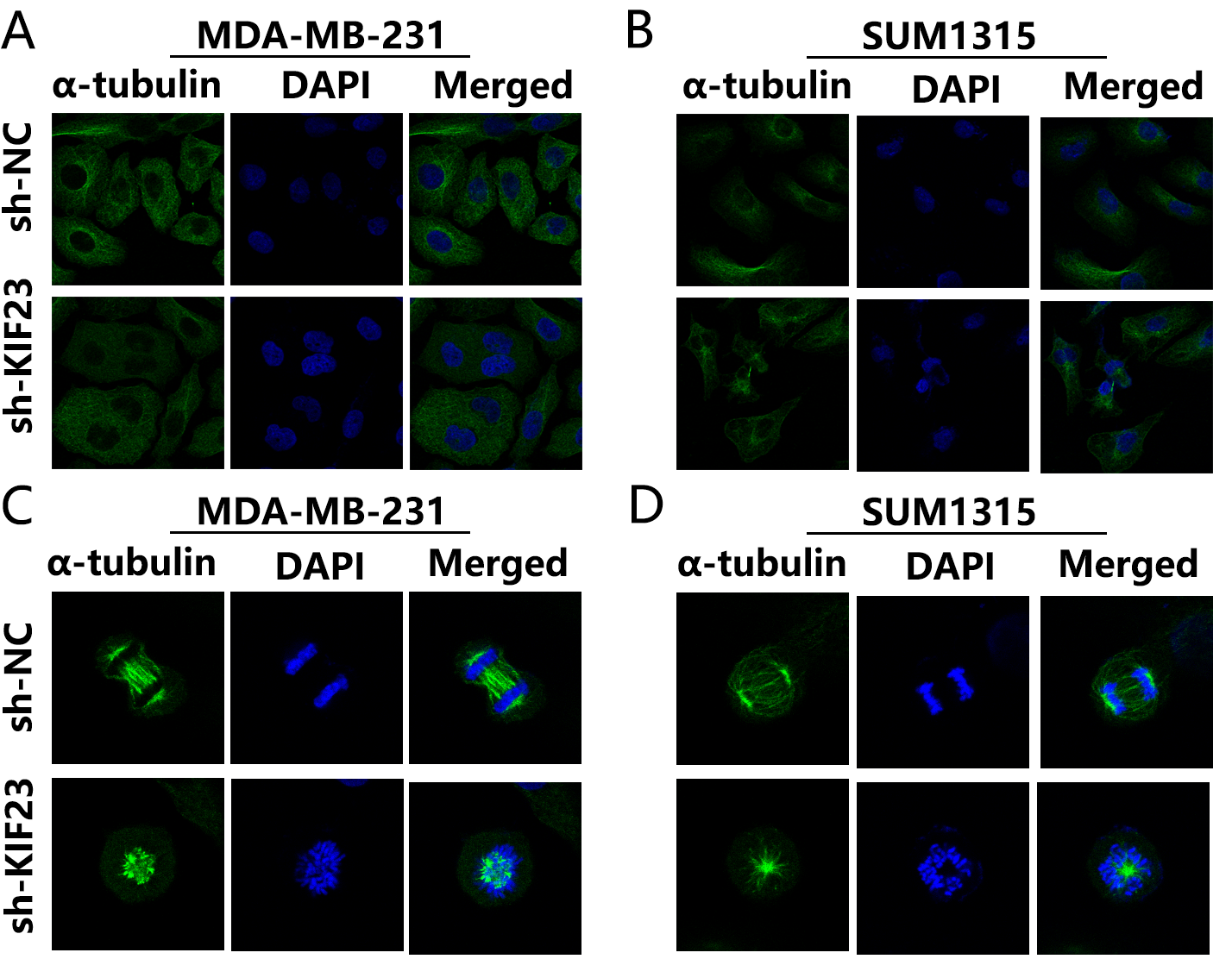

Supplement: Supplementary file 3 — Additional file 3: Figure S3. Knockdown of KIF23 causes mitotic defects in TNBC cells. [file 13046_2022_2373_MOESM3_ESM.tif]

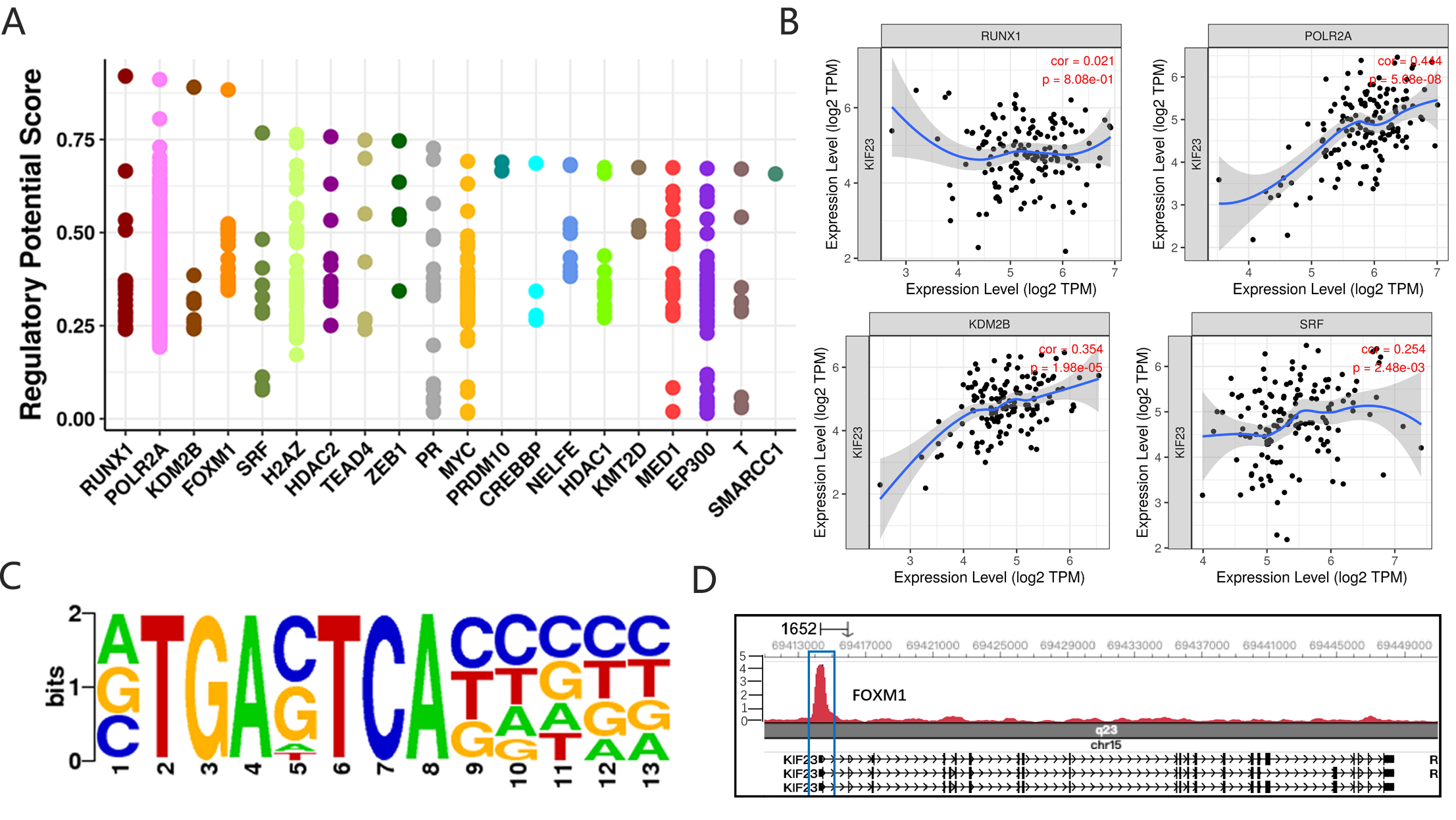

Supplement: Supplementary file 4 — Additional file 4: Figure S4. FOXM1 regulates KIF23 by binding the promoter region of KIF23. [file 13046_2022_2373_MOESM4_ESM.tif]

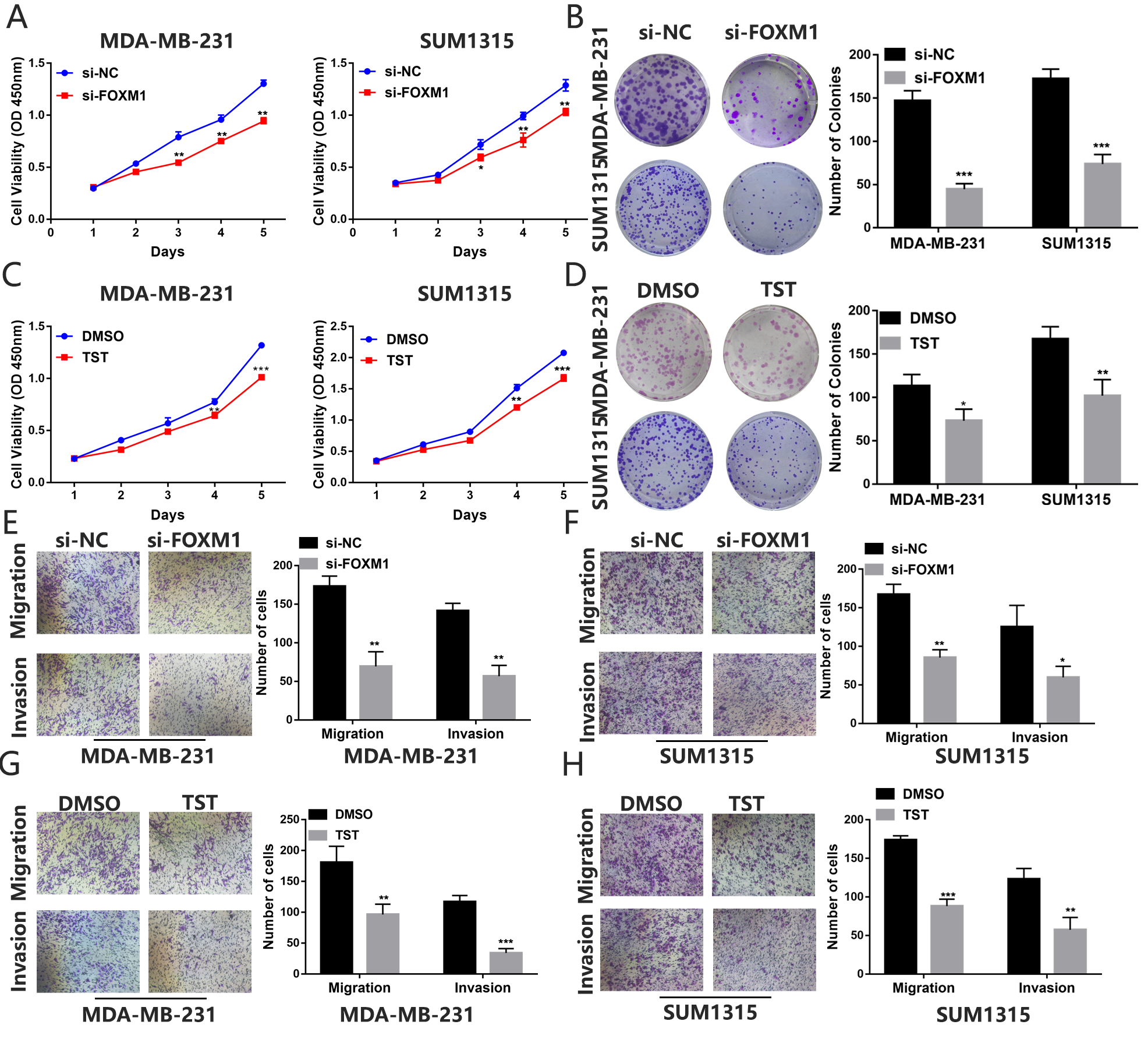

Supplement: Supplementary file 5 — Additional file 5: Figure S5. Inhibition of FOXM1 represses TNBC cell proliferation, migration and invasion. [file 13046_2022_2373_MOESM5_ESM.tif]

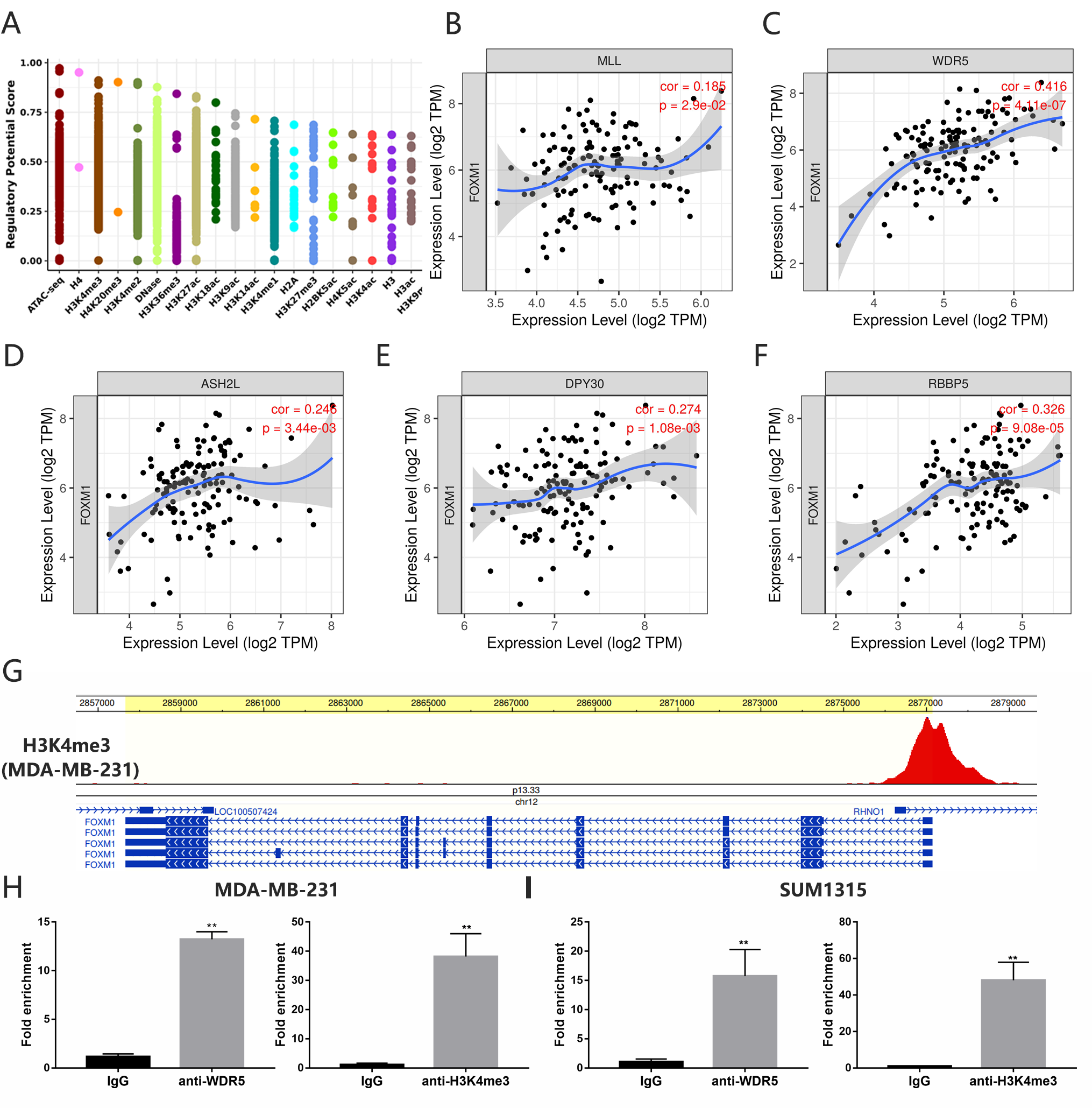

Supplement: Supplementary file 6 — Additional file 6: Figure S6. WDR5 regulates FOXM1 expression via H3K4me3 modification. [file 13046_2022_2373_MOESM6_ESM.tif]

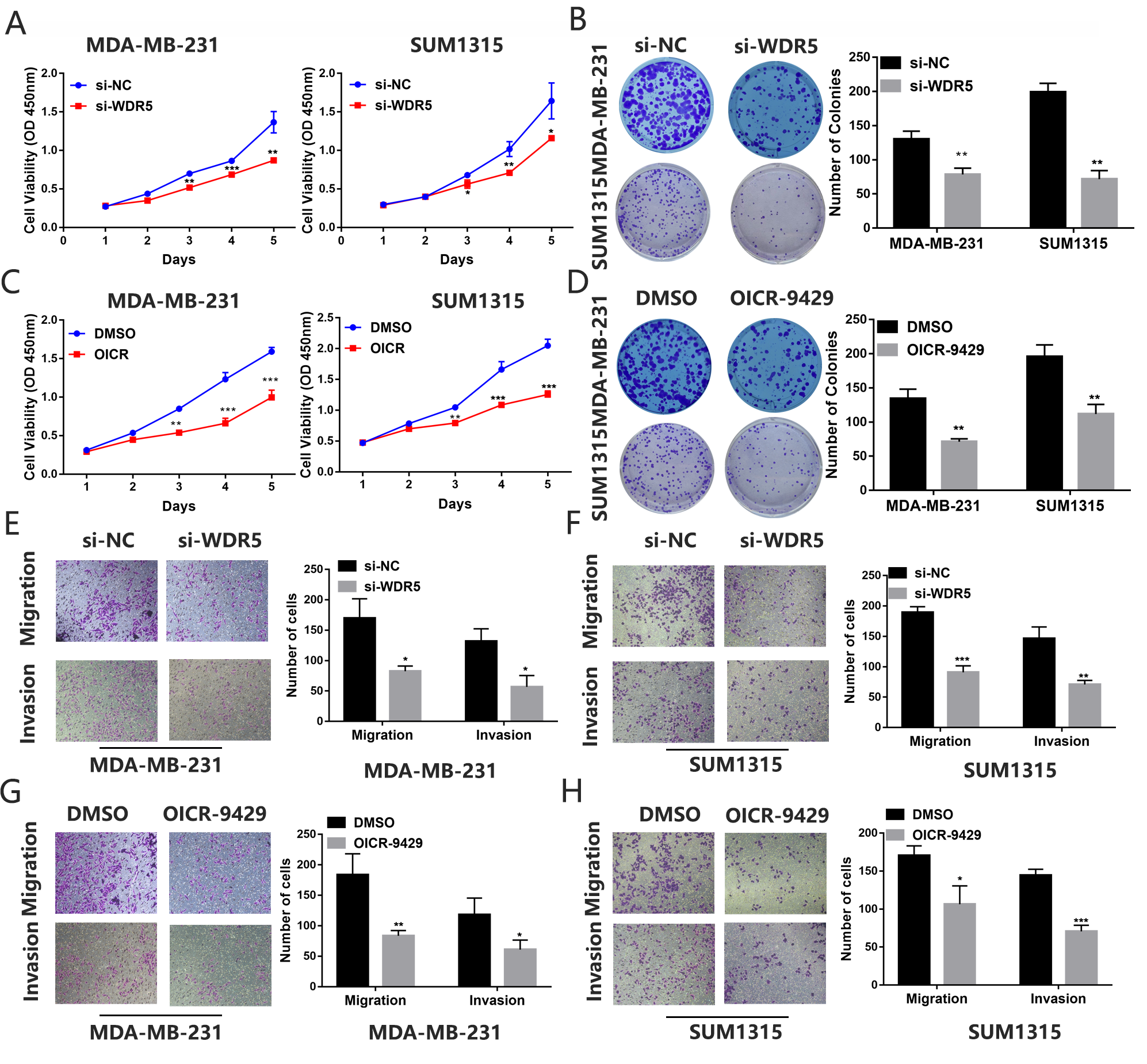

Supplement: Supplementary file 7 — Additional file 7: Figure S7. Inhibition of WDR5 represses TNBC cell proliferation, migration and invasion. [file 13046_2022_2373_MOESM7_ESM.tif]

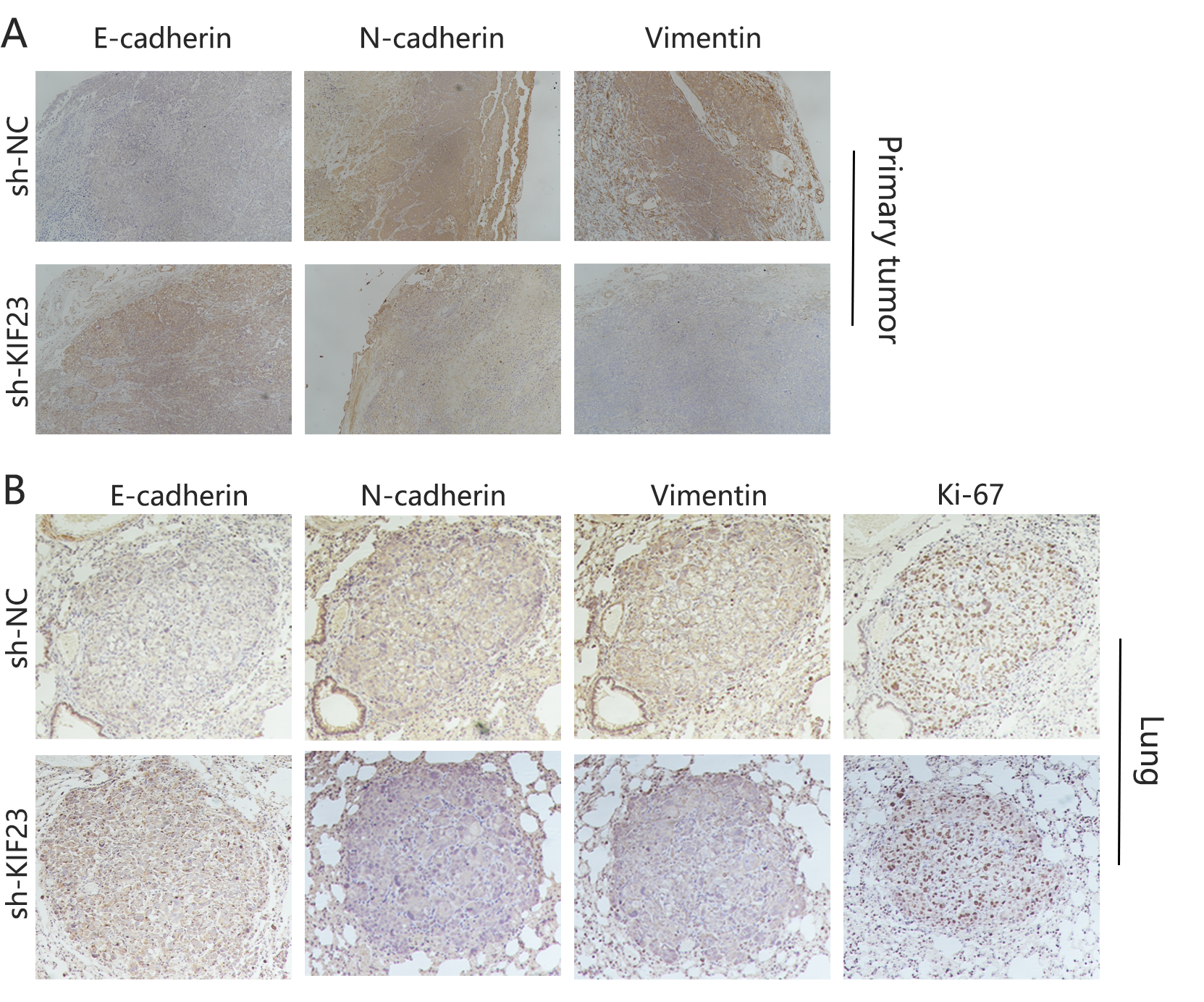

Supplement: Supplementary file 8 — Additional file 8: Figure S8. Knockdown of KIF23 reduces EMT markers and Ki-67 expression in vivo. [file 13046_2022_2373_MOESM8_ESM.tif]

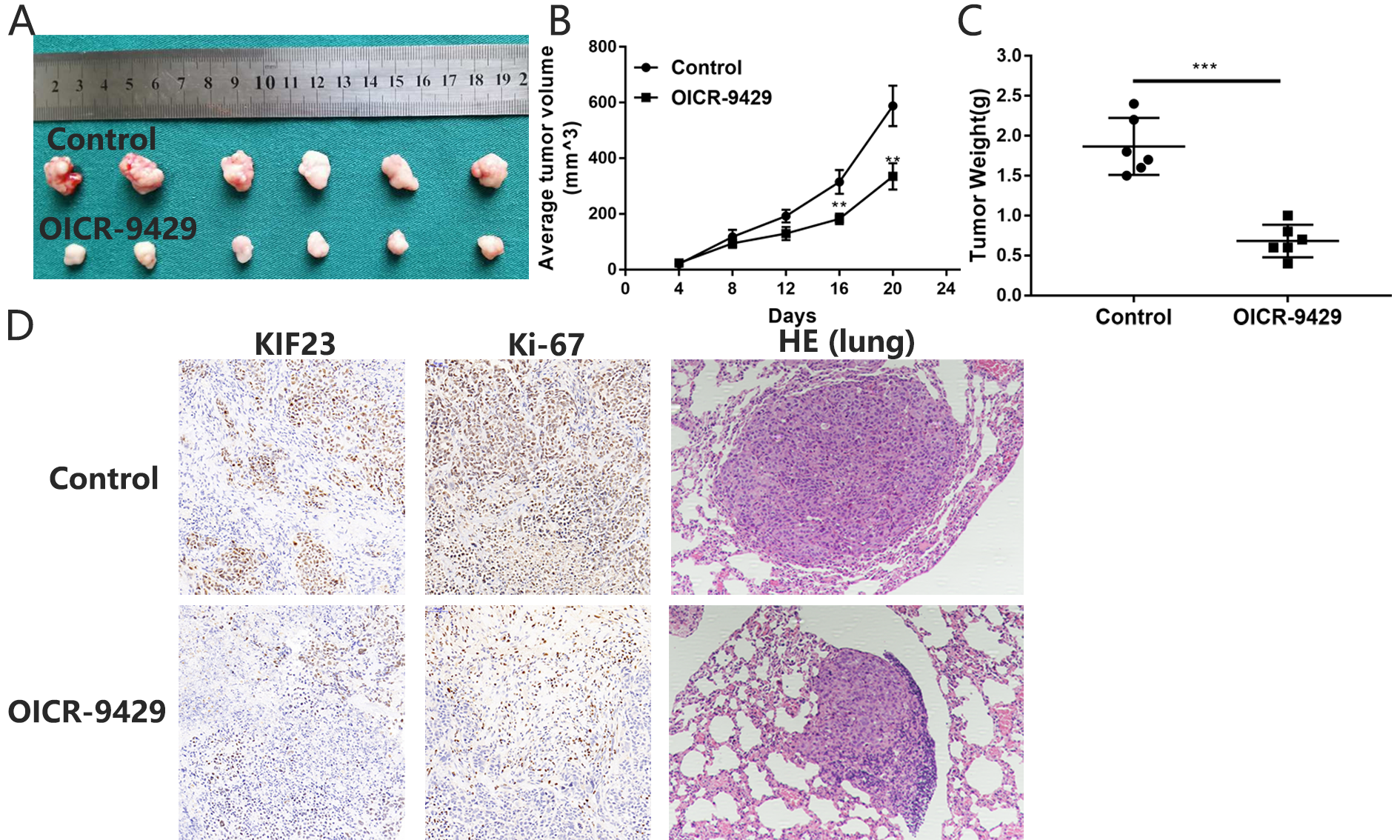

Supplement: Supplementary file 9 — Additional file 9: Figure S9. OICR-9429 treatment inhibits TNBC progression in vivo. [file 13046_2022_2373_MOESM9_ESM.tif]
