# Supplementary material for: Kinesin family member 23, regulated by FOXM1, promotes triple negative breast cancer progression via activating Wnt/β-catenin pathway
Source: J Exp Clin Cancer Res. 2022 May 7;41:168. doi: 10.1186/s13046-022-02373-7 (PMC9077852; doi:10.1186/s13046-022-02373-7)
Supplement: Supplementary file 10 — Additional file 10: Table S1. The primer sequence of qRT-PCR and CHIP analysis. Table S2. siRNA and RNA oligonucleotides sequences. [file 13046_2022_2373_MOESM10_ESM.docx]

**Supplement Table 1: The primer sequence of qRT-PCR and CHIP analysis**

| Name | Sense（5’-3’） |
| --- | --- |
| MKLP1 | F:5’-CACACCACCCAGAAGGAACT-3’  R:5’-TTTGGATTGGGCATAGCTTC-3’ |
| FOXM1 | F:5’-GAGAGGAAGCGCATGACTTT-3’  R:5’-GTCAAGTAGCGGTTGGCACT-3’ |
| WDR5 | F:5’-GGTGGGAAGTGGATTGTGTC-3’  R:5’-CAGCAGAGGCGATGATGTT-3’ |
| GAPDH | F:5’- GAAGGTGAAGGTCGGAGTC -3’  R:5’- GAAGATGGTGATGGGATTTC -3’ |
| MKLP1-CHIP | F:5’- CCTAACGTCCCGCAGTCTT -3’  R:5’- GCCTCGTACTCACGCTGAC -3’ |
| FOXM1-CHIP | F:5’-AGCCCGGAATGCCGAGACAA-3’  R:5’- GGCACCGGAGCTTTCAGTTTG-3’ |

**Supplement Table 2: siRNA and RNA oligonucleotides sequences.**

| Name | Sense（5’-3’） |
| --- | --- |
| si-NC | UUCUCCGAACGUGUCACGUTT |
| si-FOXM1 | GCUGGGAUCAAGAUUAUUATT |
| si-WDR5 | GCUCAGAGGAUAACCUUGUTT |
| Sh-MKLP1 | GGTCCCAAACGAACCTTAA |
